# Determining the Replication Kinetics and Cellular Tropism of Influenza D Virus on Primary Well-Differentiated Human Airway Epithelial Cells

**DOI:** 10.3390/v11040377

**Published:** 2019-04-24

**Authors:** Melle Holwerda, Jenna Kelly, Laura Laloli, Isabel Stürmer, Jasmine Portmann, Hanspeter Stalder, Ronald Dijkman

**Affiliations:** 1 Institute of Virology and Immunology, 3012 Bern, Switzerland; melle.holwerda@vetsuisse.unibe.ch (M.H.); jenna.kelly@vetsuisse.unibe.ch (J.K.); laura.laloli@vetsuisse.unibe.ch (L.L.); isabel.stuermer@vetsuisse.unibe.ch (I.S.); jasmine.portmann@vetsuisse.unibe.ch (J.P.); hanspeter.stalder@vetsuisse.unibe.ch (H.S.); 2 Institute of Virology and Immunology, 3147 Mittelhäusern, Switzerland; 3 Department of Infectious diseases and Pathobiology, Vetsuisse Faculty, University of Bern, 3012 Bern, Switzerland; 4 Graduate School for Cellular and Biomedical Sciences, University of Bern, 3012 Bern, Switzerland

**Keywords:** influenza D virus, zoonosis, human respiratory epithelium

## Abstract

Influenza viruses are notorious pathogens that frequently cross the species barrier with often severe consequences for both animal and human health. In 2011, a novel member of the *Orthomyxoviridae* family, Influenza D virus (IDV), was identified in the respiratory tract of swine. Epidemiological surveys revealed that IDV is distributed worldwide among livestock and that IDV-directed antibodies are detected in humans with occupational exposure to livestock. To identify the transmission capability of IDV to humans, we determined the viral replication kinetics and cell tropism using an in vitro respiratory epithelium model of humans. The inoculation of IDV revealed efficient replication kinetics and apical progeny virus release at different body temperatures. Intriguingly, the replication characteristics of IDV revealed higher replication kinetics compared to Influenza C virus, despite sharing the cell tropism preference for ciliated cells. Collectively, these results might indicate why IDV-directed antibodies are detected among humans with occupational exposure to livestock.

## 1. Introduction

After the initial discovery of Influenza D virus (IDV) in 2011, among swine with Influenza-like symptoms, knowledge about this new genus in the family of *Orthomyxoviridae* is increasing [1,2]. Epidemiological studies have shown that the virus has a worldwide distribution, whereby at least two distinct genetic lineages are cocirculating and reassorting [3,4,5,6,7,8,9,10]. Because of the high seroprevalence, cattle is the proposed natural reservoir of IDV, in which IDV causes mild respiratory disease symptoms [11]. In addition to cattle, IDV-specific antibodies have been detected in swine, feral swine, equine, ovine, caprine and camelid species, suggesting a broad host tropism for IDV [3,4,9,12,13,14]. However, the most striking observation is the detection of IDV-directed antibodies among humans with occupational exposure to livestock [15].

There are several indicators that IDV has a zoonotic potential. For instance, the utilization of the 9-*O*-acetyl-*N*-acetylneuraminic acid as a receptor determinant, that allows the hemagglutinin esterase fusion (HEF) glycoprotein of IDV to bind the luminal surface of the human respiratory epithelium [16]. Interestingly, the utilization of this receptor is also described for the closely related Influenza C virus (ICV) [17]. Furthermore, the detection of IDV-directed antibodies among humans with occupational exposure to livestock and the molecular detection of IDV in a nasopharyngeal wash of a field worker with close contact to livestock indicates that cross species transmission occurs [15,18]. However, thus far, there is no indication of wide spread prevalence among the general population although the virus has been detected during molecular surveillance of aerosols collected at an international airport [19,20]. Therefore, it remains unclear whether IDV can indeed infect cells within the human respiratory tract and, thus, whether it has a zoonotic potential.

The respiratory epithelium is the main entry port for respiratory pathogens and is, therefore, an important first barrier for intruding viruses. For more than 15 years, the human well-differentiated airway epithelial cell (hAECs) culture model has been applied as an in vitro surrogate model of the in vivo respiratory epithelium to investigate a wide range of emerging and zoonotic respiratory viruses on their capability of direct transmission to humans [21,22,23,24,25].

The aim of this study is to investigate the transmission capability of IDV to humans by inoculating well-differentiated hAEC cultures with IDV. In addition, we sequentially passaged IDV further on naïve well-differentiated hAEC cultures to determine whether infectious progeny virus is produced. This revealed that IDV is able to efficiently replicate in well-differentiated hAEC cultures and can be passaged subsequently without extensive genetic adaptations. Moreover, due to the similarity of IDV with ICV, we compared their viral kinetics and cell tropism. This showed that IDV has higher replication kinetics compared to ICV, and that both viruses share a cell tropism preference towards ciliated cells. These results emphasize that there is no fundamental restriction of IDV replication within the human respiratory epithelium. Therefore, these findings might explain why IDV-specific antibodies can be detected in humans with occupational exposure to livestock.

## 2. Materials and Methods

### 2.1. Cell Culture

The Madin–Darby Bovine Kidney (MDBK) cells were maintained in Eagle’s minimum essential medium (EMEM; (Gibco, Gaithersburg, MD, USA) supplemented with 7% heat-inactivated fetal bovine serum (FBS, Gibco), 2 mmol/L Glutamax (Gibco), 100 µg/mL Streptomycin and 100 IU/mL Penicillin (Gibco). Human rectal tumor 18G (HRT-18G) cells (ATCC, Manassas, VA, USA) were maintained in Dulbecco’s minimum essential medium (DMEM; Gibco) supplemented with 5% heat-inactivated FBS, 100 µg/mL Streptomycin and 100 IU/mL Penicillin (Gibco). Both cell lines were propagated at 37 °C in a humidified incubator with 5% CO_2_.

### 2.2. Viruses

For the initial characterization experiments, Influenza D virus (D/bovine/Oklahoma/660/2013) was inoculated on MDBK cells and propagated in infection medium (EMEM, supplemented with 0.5% bovine serum albumin (Sigma-Aldrich, St. Louis, MO, USA), 15 mmol/L of HEPES (Gibco), 100 µg/mL Streptomycin and 100 IU/mL penicillin (Gibco), and 1 μg/mL bovine pancreas-isolated acetylated trypsin (Sigma-Aldrich)). Infected MDBK cultures were maintained for 96 h at 37 °C. For the direct comparison of IDV (D/bovine/Oklahoma/660/2013) and ICV (C/Johannesburg/1/66), both viruses were inoculated on HRT-18G cells and propagated for 96 h at 37 °C or 33 °C, respectively, in infection medium supplemented with 0.25 μg/mL Bovine pancreas-isolated acetylated trypsin. Virus containing supernatant was cleared from cell debris through centrifugation for 5 min at 500× *rcf* before aliquoting and storage at −80 °C.

### 2.3. Human Airway Epithelial Cell (hAEC) Culture

Primary human bronchial cells were isolated from patients (>18 years old) undergoing bronchoscopy or pulmonary resection at the Cantonal Hospital in St. Gallen, Switzerland, in accordance with our ethical approval (EKSG 11/044, EKSG 11/103 and KEK-BE 302/2015). Isolation and culturing of primary human bronchial epithelial cells was performed as previously described [26,27], with the minor modification of supplementing the BEGM with 10 µmol/L Rho associated protein kinase inhibitor (Y-27632, Abcam, Cambridge, UK).

### 2.4. Viral Replication in Well-Differentiated hAEC Cultures

Well-differentiated hAEC cultures were inoculated with 10,000 tissue culture infectious dosis 50 (TCID_50_) of either IDV or ICV. The viruses where incubated for 1.5 h at temperatures indicated in a humidified incubator with 5% CO_2_. Afterwards, inoculum was removed, and the apical surface was washed thrice with Hanks balanced salt solution (HBSS, Gibco), after which the cells were incubated at the indicated temperatures in a humidified incubator with 5% CO_2_. The infection was monitored as previously described, during which progeny virus was collected by incubating the apical surface with 100 μL HBSS 10 min prior to the time point. Collected apical washes were stored 1:1 in virus transport medium for later quantification [27].

### 2.5. Virus Titration by Tissue Culture Infectious Dosis 50 (TCID_50_)

MDBK cells were seeded at a concentration of 40,000 cells per well in a 96-well cluster plates, whereas HRT-18G cells were seeded at a concentration of 100,000 cells per well. The following day, medium was removed, and cells were washed once with PBS and replaced with 50 μL of infection medium. Virus containing samples were 10-fold serial diluted in infection medium, from which 50 μL was added to the target cells in six technical replicates per sample. For MDBK, the inoculated cells were incubated for 72 h at 37 °C in a humidified incubator with 5% CO_2_, where after they were fixed by crystal violet to determine the viral titer. The HRT-18G cells were incubated for 120 h at 33 °C or 37 °C, for ICV and IDV respectively, in a humidified incubator with 5% CO_2_, where after 50 µL of supernatant was used as input for an hemagglutination assay, as described below, to determine the viral titer. The viral titer was calculated according to the protocol of Spearman-Kärber [28].

### 2.6. Hemaglutination Assay

Chicken blood for the hemagglutination agglutination (HA) and hemagglutination inhibition (HI) assays was obtained from SPF-bred white Leghorn chickens in compliance with the Animal Welfare Act (TSchG SR 455), the Animal Welfare Ordinance (TSchV SR 455.1), and the Animal Experimentation Ordinance (TVV SR 455.163) of Switzerland. That was reviewed by the ethical committee for animal experiments of the canton of Bern and approved by the cantonal veterinary authorities (Amt für Landwirtschaft und Natur LANAT, Veterinärdienst VeD, Bern, Switzerland) with the agreement BE78/17. The HA assays were performed using 1% chicken red blood cells diluted in ice-cold PBS as described previously [29]. For the HI assay, Intravenous Immunoglobulins (IVIg; Sanquin, The Netherlands) was pretreated with receptor-destroying enzyme (Denka Seiken, Tokyo, Japan) for 18 h at 37 °C, followed by an inactivation for 30 min at 56 °C. The HA- or HI-titer was determined after 30 min incubation at room temperature by recording the highest serial dilution that still displayed tear-formation after the plate was tilted 45° for 30 s. According to the WHO protocol guidelines a HI titer of <10 was regarded as negative [29].

### 2.7. Quantitative Real-Time Reverse Transcription Polymerase Chain Reaction (PCR)

For quantification of the viral kinetics of IDV and ICV, viral RNA was extracted from 50 µL apical wash using the NucleoMag VET (Macherey-Nagel AG, Oensingen, Switzerland), according to the manufacturer’s guidelines, on a Kingfisher Flex Purification system (Thermo Fisher Scientific, Darmstadt, Germany). Two microliters of extracted RNA was amplified using TaqMan™ Fast Virus 1-Step Master Mix (Thermo Fisher Scientific) according to the manufacturer’s protocol using the forward primer 5′-AACCTGCTTCTGCTTGCAATCT-3′, reverse 5′-AACAATGAACAGTTACCGCATCA-3′ and probe 5′-FAM-AGACCTGTCTAAAACTATTT-BHQ1-3′ targeting the P42-segment of ICV (AM410042.1). Whereas for the P42-segment of IDV (KF425664.1) the forward 5′-ATGCTGAAACTGTGGAAGAATTTTG-3′, reverse 5′-GGTCTTCCATTTATGATTGTCAACAA-3′ and probe 5′-FAM-AAGGTTTATGTCCATTGTTTCA-BHQ1-3′ were used. A standard curve of the P42-segment of Influenza C or D virus, cloned in pHW2000 plasmid, was included to interpolate the amount of genomic equivalents [30]. Measurements and analysis were performed using an ABI7500 instrument and software package (Applied Biosystems, Foster City, CA, USA).

### 2.8. Antibody Specificity of Influenza D Virus (IDV) Nucleoprotein (NP) Antibody

MDBK cells were seeded at a density of 100,000 cells on 0.17 mm high precision coverslips (Marienfeld, Lauda-Königshofen, Germany) in a 24-well cluster plate. The following day, cells were infected with IDV at a multiplicity of infection (MOI) of 0.1 as previous described for a duration of 24 h. Afterwards cells were formalin-fixed and stained for immunofluorescence as previously described [27]. Mock and IDV-infected cultures were incubated with either the custom generated rabbit polyclonal antibody directed against the nucleoprotein (NP) of the prototypic D/bovine/Oklahoma/660/2013 strain (Genscript, Piscataway, NJ, USA) or the corresponding pre-immunization rabbit serum. Followed by applying Alexa Fluor^®^ 647-labeled donkey anti-Rabbit IgG (H + L) (Jackson Immunoresearch, Westgrove, PA, USA) or Alexa Fluor^®^ 647-labeled donkey anti-Mouse IgG (H + L) as secondary antibody. For certain samples either the primary or secondary antibodies were omitted during the incubation steps. All samples were counterstained using 4’,6-diamidino-2-phenylindole (DAPI, Thermo Fisher Scientific) to visualize the nuclei. The immunostained coverslips were mounted on Colorforst Plus microscopy slides (Thermo Fisher Scientific) in Prolong diamond antifade mountant (Thermo Fisher Scientific). Microscopic images were acquired on a DeltaVision Elite High-Resolution imaging system (GE Healthcare Life Sciences, Chicago, IL, USA) with a 60×/1.42 oil objective. Images were processed using the Imaris version 9.1.3 (Bitplane AG, Zurich, Switzerland) software package.

### 2.9. Immunofluorescence of Well-Differentiated hAEC Cultures

Well-differentiated hAEC cultures were formalin-fixed and stained for immunofluorescence as previously described [27]. For the detection of IDV-positive cells, hAEC cultures were stained with a custom-generated rabbit polyclonal antibody directed against the NP of the prototypic D/bovine/Oklahoma/660/2013 strain (Genscript). Alexa Fluor^®^ 647-labeled donkey anti-Rabbit IgG (H + L) (Jackson Immunoresearch) was applied as secondary antibody. For the characterization and quantification of the cell tropism, hAEC cultures were stained with the custom-generated polyclonal rabbit anti-NP (Genscript), mouse Anti-β-tubulin IV (AB11315, Abcam) and goat anti-ZO1 (AB99642, Abcam). Alexa Fluor^®^ 488-labeled donkey anti-mouse IgG (H + L), Cy3-labeled donkey anti-goat IgG (H + L) and Alexa Fluor^®^ 647-labeled donkey anti-Rabbit IgG (H + L) (Jackson Immunoresearch) were used as secondary antibodies. In the case of ICV, hAEC cultures were stained with human IVIg (Sanquin, Amsterdam, The Netherlands), mouse Anti-β-tubulin IV (AB11315, Abcam) and rabbit anti-ZO1 (617300, Thermo Fisher Scientific). Using Alexa Fluor^®^ 488-labeled donkey anti-mouse IgG (H + L), Alexa Fluor^®^ 594-labeled donkey anti-human IgG (H + L) and Alexa Fluor^®^ 647-labeled donkey anti-Rabbit IgG (H + L) (Jackson Immunoresearch) as secondary antibodies. All samples were counterstained using 4’,6-diamidino-2-phenylindole (DAPI, Thermo Fisher Scientific) to visualize the nuclei. The immunostained inserts were mounted on Colorforst Plus microscopy slides (Thermo Fisher Scientific) in Prolong diamond antifade mountant (Thermo Fisher Scientific) and overlaid with 0.17 mm high precision coverslips (Marienfeld). The Z-stack images were acquired on a DeltaVision Elite High-Resolution imaging system (GE Healthcare Life Sciences) using a step size of 0.2 µm with a 60×/1.42 oil objective. Images were deconvolved and cropped using the integrated softWoRx software package and processed using Fiji [31] and Imaris version 9.1.3 (Bitplane AG, Zurich, Switzerland) software packages.

### 2.10. Deep Sequencing Analysis of IDV Passaged upon Well-Differentiated hAEC Cultures

To analyze potential genetic alterations during the passaging of IDV on well-differentiated hAEC cultures, we extracted the RNA as described above. Two microliters of RNA was used as templates to amplify of each of the 7 individual genomic segments with segment specific primers (Table 1) using Superscript IV one-step reverse transcription polymerase chain reaction (RT-PCR) kit (Thermo Fisher Scientific), according to manufacturer guidelines. For each sample the amplified segments were pooled together at equal molar concentrations in order to reach a uniform sequencing depth for each individual genomic segment. The sequencing libraries of the pooled amplified segments were prepared using the Nextera XT library kit (Illumina, San Diego, CA, USA) and sequenced on a MiSeq Illumina platform using 300 bp paired-end reads, according to manufactures protocol. The raw MiSeq Illumina reads were first trimmed using Trim Galore! (version 0.4.5) and then FastQC (version 0.11.7) was used to assess the overall read quality [32]. Trimmed reads from the inoculum virus sample were then aligned against the D/bovine/Oklahoma/660/2013 reference sequence (GenBank: KF425659–KF425665) using the Bowtie2 aligner (version 2.3.4.1) and Samtools (version 1.8) was used to generate a consensus sequence from this alignment [33,34]. The inoculum virus consensus sequence was then used as a reference to align the processed reads from the passage 3 viruses that were cultured at both 33 °C and 37 °C. Nucleotide variants were called using Lofreq (version 2.1.2) and supplemented with the gene feature metadata of D/bovine/Oklahoma/660/2013 using SnpEff (version 4.3) [35,36]. Sequencing depth for the genomic segments in each sample were analyzed with Samtools (version 1.8). Calculations were performed on UBELIX (http://www.id.unibe.ch/hpc), the High Performance Computing (HPC) cluster at the University of Bern.

### 2.11. Data Presentation

Titration and replication kinetics data were plotted using GraphPad Prism 8 (San Diego, CA, USA). The obtained information from the deep sequencing analysis was imported into R (version 3.5.2) in order to visualize the sequencing depth and summarize the nucleotide variants using a number of packages in R, including Tidyverse (version 1.2.1), reshape2 (version 1.4.3), scales (version 1.0.0), and ggrepel (version 0.8.0). The final figures were assembled in Adobe Illustrator CS6.

## 3. Results

### 3.1. Efficient Replication of IDV in Well-Differentiated hAEC Cultures

**Figure 1:**
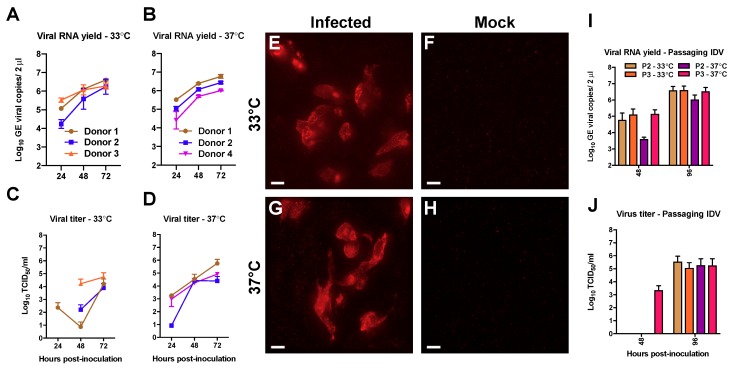
Efficient replication of Influenza D virus (IDV) in well-differentiated human airway epithelial cell (hAEC) cultures. Human airway epithelial cell cultures were inoculated with 10,000 tissue culture infectious dosis 50 (TCID_50_) of IDV and incubated at either 33 °C or 37 °C. The monitored viral RNA yield is given as genomic equivalents (GE) per 2 μL of isolated RNA (*y*-axis) at indicated hours post-inoculation (*x*-axis) for 33 °C (**A**) and 37 °C (**B**). Whereas the viral titer is given as TCID_50_/mL (*y*-axis) for 33 °C (**C**) and 37 °C (**D**) at indicated hours post-inoculation (*x*-axis). These results are displayed as means and standard deviation (SD) from duplicates from three independent donors. Human airway epithelial cell cultures were formalin-fixed and immunostained with a custom generated antibody against the nucleoprotein (NP) of Influenza D virus to detect viral antigen. A representative image from one of the three independent donors is shown for IDV infection at 33 °C and 37 °C (**E**,**G**) as well as their respective controls (**F**,**H**). Magnification 60×, the scale bar represents 10 micrometers. To assess if IDV viral progeny is infectious, hAEC cultures were inoculated with tenfold-diluted apical wash of passage 1 virus that was serial-passaged, for two consecutive passages, upon naïve hAEC cultures. The monitored viral RNA yield is given as genomic equivalents (GE) per 2 μL of isolated RNA (*y*-axis) at indicated hours post-inoculation (*x*-axis) for each of the conditions (**I**). Whereas the viral titer is given as TCID_50_/mL (*y*-axis) for each condition, at indicated hours post-inoculation (*x*-axis) (**J**). The results are displayed as means and SD from duplicates from three independent donors.

As a first step to address the transmission capability of IDV to humans we inoculated the prototypic D/bovine/Oklahoma/660/2013 strain on well-differentiated hAEC cultures of three biological donors. Viral progeny release was monitored by collecting washes with 24-h intervals for a duration of 72 h. To analyze temperature dependent effects, incubation of the cultures was performed at temperatures that correspond with those of the human upper and lower respiratory tract, 33 °C and 37 °C respectively. The release of viral progeny from the apical washes was analyzed by quantitative real-time reverse transcription PCR for viral transcripts and virus titration for an infectious virus. The first viral transcripts were detected at 24 h post-infection (hpi) among all donors, independently of the incubation temperature (Figure 1A,B). However, some temperature-dependent differences were observed when the infectivity of the progeny virus was analyzed. When incubated at 33 °C, viral titers were detected for every donor at 48 and 72 hpi, but only one donor shows a viral titer at 24 hpi (Figure 1C). In contrast, we observed viral titers as early as 24 hpi for IDV infection at 37°C for every donor that increased over time (Figure 1D). These results suggest that IDV kinetics seems to be more efficient at ambient temperatures corresponding to the human lower respiratory tract, although the differences between the viral titers at 72 hpi are minor.

After having demonstrated that IDV is able to replicate in well-differentiated hAEC cultures from different donors at both 33 °C and 37 °C, we wanted to corroborate these results via immunofluorescence analysis. However, commercial antibodies against IDV are currently unavailable, so we ordered a custom generated antibody directed against the nucleoprotein (NP) of the prototypic D/bovine/Oklahoma/660/2013 strain. After securing the NP-antibody specificity (Figure S1), the microscopic analysis of IDV-infected hAEC cultures revealed clusters of NP-positive cells at both 33 °C and 37 °C, whereas no fluorescence signal was observed in the control hAEC cultures (Figure 1E–H). The majority of the fluorescence signal from the NP-positive cells has a cytoplasmic distribution pattern, but some of those also appeared to have a nuclear staining pattern. These findings suggest that, like other Orthomyxoviruses, the NP of IDV is actively translocated to the nucleus during viral replication [37,38]. Combined, these results demonstrate that IDV is able to replicate efficiently in hAEC cultures from different donors at temperatures corresponding to both the upper and lower respiratory tract of humans.

To analyze if IDV progeny virus is able to infect naïve well-differentiated hAECs of a new donor, we sequentially passaged a 10-fold dilution of the previous obtained 72 hpi apical wash from our three donors on a naïve donor (P2). After 48 hpi, we collected the apical wash of P2 and made a 10-fold diluted that was sub-passaged upon naïve hAEC cultures of the same donor (P3). As before, we performed these experiments at both 33 °C and 37 °C to assess whether there are temperature dependent effects. We monitored the production of viral progeny at 48 and 96 hpi for each of the passages. In the first passage (P2), viral RNA was detected at 48 hpi and increased with one order of magnitude at 96 hpi (Figure 1I). However, we observed that the viral RNA yield at 37 °C was approximately one order lower in the first round compared to the viral RNA yield at 33 °C, while at 96 hpi this difference slightly reduced. Interestingly, no difference between the different incubation temperatures was observed in the final passage (P3) (Figure 1J). Also, no pronounced differences were observed in the viral titers between the different temperatures or passage numbers at 96 hpi (Figure 1J). However, at 48 hpi, we only detect infectious virus in the apical wash from the last passaging experiment that was performed at 37 °C (P3; Figure 1J). These results show that the viral progeny from the initial experiments on well-differentiated hAEC cultures is infectious and that IDV can be serial-passaged on hAEC cultures from different donors at both 33 °C and 37 °C.

### 3.2. Deep Sequencing Analysis of IDV Passaged upon Well-Differentiated hAEC Cultures

**Figure 2:**
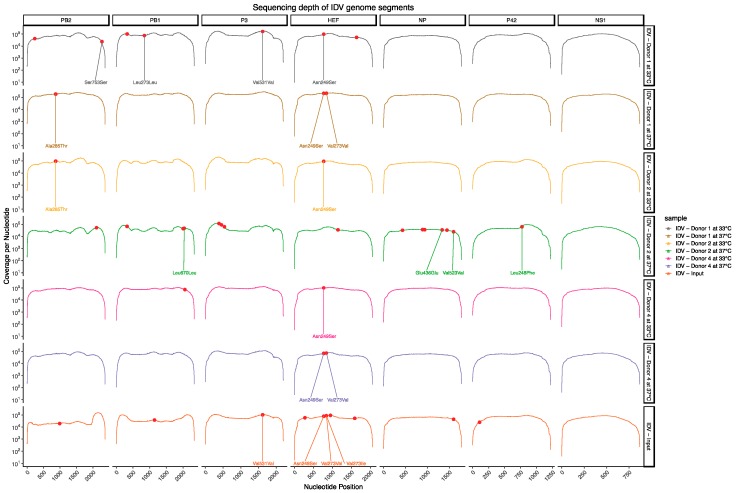
Sequencing depth of IDV genome segments. To assess eventual host adaptation during the serial-passaging of IDV on well-differentiated hAEC cultures, the complete genome of the parental inoculum and the viruses that were obtained after 3 serial-passages at 33 °C or 37 °C were amplified and sequenced. The sequence coverage per nucleotide (*y*-axis) and corresponding nucleotide position (*x*-axis) is provided for the 7 different genomic segments from the parental and serial-passaged viruses. The nucleotide variants are highlighted with a red point, whereas the resulting amino acids are only annotated for those variants with a detection frequency above 10%, as indicated in Table 2.

Because cattle are considered to be the main reservoir for IDV, we wondered whether any genetic changes might have occurred during the serial-passaging experiment of IDV on well-differentiated hAEC cultures. Since host adaptations usually arise after a few generations, we chose to only sequence the complete genome of the parental inoculum and the viruses that were obtained after 3 serial-passages at 33 °C or 37 °C. In total, we detected 33 different nucleotide variants among the different samples (Figure 2 and Table 2). Interestingly, these nucleotide variants were only detected in 6 of the 7 genomic segments. No nucleotide variants were detected for the genomic segment encoding for the NS1 and NS2 proteins. For our parental inoculum virus, we detected 11 nucleotide variants that were distinct from the D/bovine/Oklahoma/660/2013 reference sequence that is deposited in Genbank (Figure 2 and Table 2). To assess for possible host and/or temperature-dependent adaptations in the passage 3 viruses we only considered nucleotide variants that were represented in viruses obtained from at least 2 different donors for each incubation temperature. This revealed that within the coding sequence for the HEF protein the frequency of the non-synonymous variant N249S at nucleotide position 770 seems to become more prevalent in the viruses that were three times serial-passaged in well-differentiated hAEC cultures (Table 2). This seems to have occurred independently from the temperature at which the viruses were propagated (Table 2). Interestingly, at nucleotide position 841 in the HEF gene segment we detected two genetic variants in the parental inoculum virus, which leads respectively to a non- or synonymous variant V273I and V273V (Table 2). The non-synonymous V273I variant has the highest prevalence, however it is absent among four of the six passaged virus isolates. Indicating that these isolates have reverted back to the corresponding D/bovine/Oklahoma/660/2013 reference sequence in Genbank (Table 2), whereas among the two remaining virus isolates the synonymous V273V variant dominates (Figure 2 and Table 2). This shows that in humans, as well as all of the current known isolates from cattle and swine, the valine amino acid at position 273 is favored over an isoleucine. In contrast to these changes, we observed only one non-synonymous change at position N249S that seems to be a potential adaptation of IDV to well-differentiated hAEC cultures (Figure 2 and Table 2). Although, to what extent this non-synonymous N249S variant influences the replication kinetics of IDV on well-differentiated hAEC cultures remains to be addressed. Nonetheless, the deep sequencing results highlight that despite multiple passages there is most likely minimal adaptation required for IDV to replicate efficiently in the human respiratory epithelium.

### 3.3. Comparison of Influenza C Virus (ICV) and IDV Infection in Well-Differentiated hAEC Cultures

**Figure 3:**
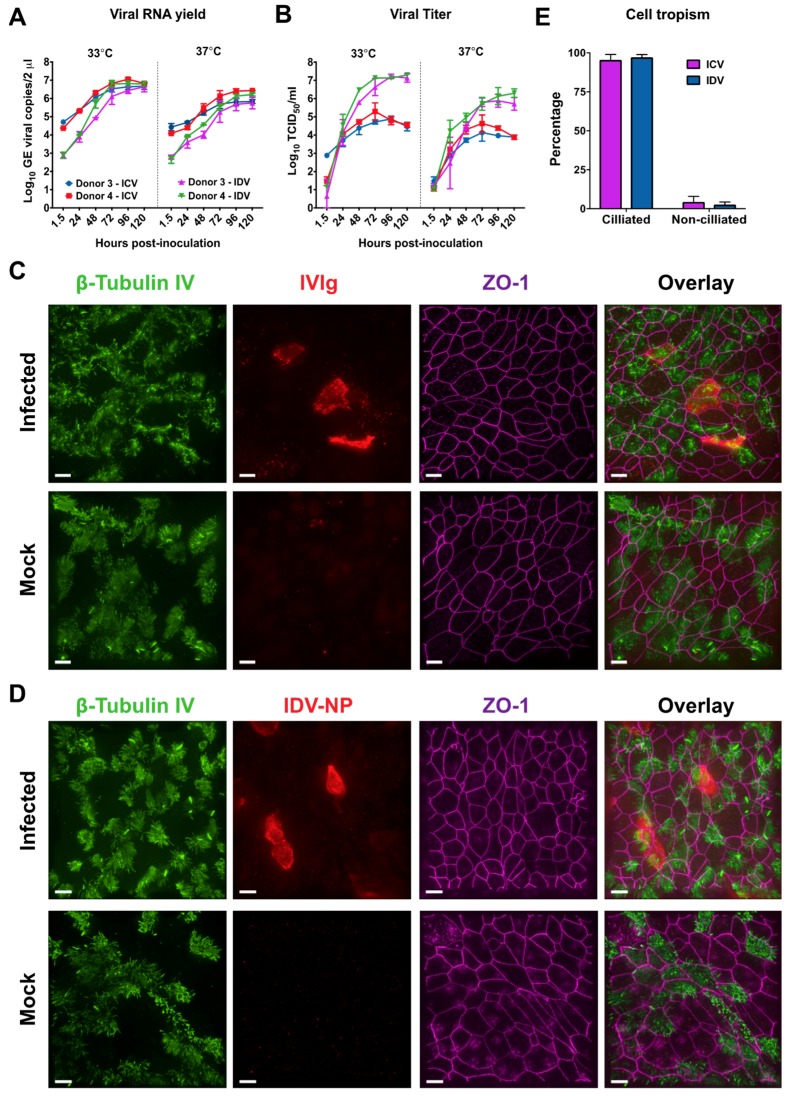
Comparison of Influenza C virus (ICV) and IDV infection in well-differentiated hAEC cultures. Human airway epithelial cell cultures were inoculated with 10,000 TCID_50_ of ICV or IDV and incubated at 33 °C and 37 °C. The monitored viral RNA yield is given as genomic equivalents (GE) per 2 μL of isolated RNA (*y*-axis) at indicated hours post-inoculation (*x*-axis) for ICV and IDV (**A**). The viral titers of the corresponding apical washes are given as TCID_50_/mL at indicated hours post-infection (*x*-axis) (**B**). The results are displayed as means and SD from duplicates from two independent donors. Formalin-fixed ICV and IDV infected well-differentiated hAEC cultures and their respective controls were immunostained with antibodies to visualize the cilia (β-tubulin IV, green), tight junction borders (ZO-1, purple). Whereas virus-infected cells (red) were visualized with either a custom generated IDV NP-antibody or intravenous immunoglobulins (IVIg) for ICV (**C**&**D**). Magnification 60×, the scale bar represents 10 micrometers. The cell tropism of ICV (purple bars) and IDV (blue bars) was quantified by calculating the percentage of viral antigen-positive signal co-localization with either ciliated or non-ciliated cells (**E**). The mean percentage and SEM from ten random fields from three independent donors are displayed.

Influenza C virus is a well-known common cold virus that is able to cause a mild upper respiratory tract infection in humans [39,40]. Because of this property and the structural similarity between the HEF of IDV and ICV, we wondered how IDV replication efficiency relates to ICV in our well-differentiated hAEC cultures [16]. To address this question, we first sought to limit any potential experimental variance between the readout of the viral titers. This was done by calibrating the virus stock production and viral titration of ICV (C/Johannesburg/1/66) and IDV (D/bovine/Oklahoma/660/2013) on a single cell line, namely HRT-18G. For the direct comparison of both viruses we inoculated well-differentiated hAEC cultures of two human donors with 10,000 TCID_50_ of ICV or IDV and incubated the cultures at both 33 °C and 37 °C. This was done to determine whether there are any temperature-dependent replication kinetic differences between ICV and IDV. The viral replication kinetics were monitored as before, by collecting apical washes every 24 h, however this time for a duration of 120 h instead of 72. This was done because previously we only observed minor replication kinetic difference for IDV between 33 °C and 37 °C. We observed that, after removing the inoculum and extensive washing, the viral RNA yield increases over time for both viruses at 33 °C and 37 °C (Figure 3A). However, during the first 24 h the viral RNA yield of ICV is approximately a 1–1.5 order of magnitude higher compared to IDV, but in the end reaches the same plateau level (Figure 3A). For both viruses, the viral RNA yield plateau at 33 °C is approximately one order of magnitude higher compared to 37 °C (Figure 3A). Interestingly, when we analyzed the viral titer of the corresponding samples, a gradual increase can be observed for ICV that reaches a plateau at 72 h. In contrast to ICV, we observed a steep increase at both 33 °C and 37 °C for IDV that reaches a plateau between 72–96 hpi. The steep increase results in a pronounced difference of 2 to 3 orders of magnitude difference in the production of infectious progeny virus between ICV and IDV (Figure 3B). This difference seems to be independent of the incubation temperature and clearly demonstrates that IDV replication on well-differentiated hAEC cultures is more efficient compared to ICV (Figure 3B).

In addition to the replication kinetics, we wanted to determine the respective cell tropism for ICV and IDV, as both viruses utilize 9-*O*-acetyl-*N*-acetylneuraminic acids as receptor determinant [16,17]. Therefore, we formalin-fixed the infected hAEC cultures to determine the cell tropism for both viruses via immunostaining. To discriminate between the ciliated and non-ciliated cell types, we used well-defined antibodies to visualize the cilia (β-tubulin IV) and tight junction borders (Zonula occludens-1, ZO-1). We used our IDV-NP-antibody for detection of IDV-infected cells, whereas for ICV we used the commercially available pooled human intravenous immunoglobulins (IVIg). We used IVIg since most people have encountered one or multiple ICV infections during their life and therefore this likely contains antibodies directed to ICV [39,40]. This we corroborated by an hemagglutination inhibition assay using IVIg (Figure S2). By overlaying the different cellular marker stains with that of the virus antigen, we observed that for both ICV and IDV the virus-antigen signal overlaps with that of the ciliated cell marker (Figure 3C,D).

To accurately define the cell tropism, we counted all cell types among ten random fields per donor, with the criteria of at least having one virus-positive cell. For the IDV-infected hAEC cultures, we counted a total of 2273 cells from which 94 were NP-positive, while for ICV a total of 2526 cells and 84 ICV antigen-positive cells was observed (Table 3). The majority of antigen-positive cells for both IDV and ICV overlapped with the ciliated cell marker with an overall percentage of 97.3 and 95.5, respectively (Figure 3E). This is in line with our initial observation and shows that both IDV and ICV have a predominant preference for ciliated cells. In addition to the cellular tropism, we also calculated the overall infection rate for IDV and ICV, which is 4.3% and 3.3%, respectively. The slightly higher infection rate of IDV compared to ICV seems to be in accordance with the previous observed differences in replication kinetics (Figure 3A,B).

Collectively, our results demonstrate that IDV replication kinetics in well-differentiated hAEC cultures are much more efficient compared to ICV, despite sharing a cell tropism preference towards ciliated cells. Most importantly, these results show that there is no intrinsic impairment of IDV propagation within the human respiratory epithelium.

## 4. Discussion

In this study, we demonstrate that IDV replicates efficiently in an in vitro surrogate model of the in vivo respiratory epithelium at ambient temperatures that correspond to the human upper and lower respiratory tract. We also demonstrate that IDV viral progeny is replication competent, as it can be efficiently sequentially propagated onto well-differentiated hAEC cultures from different donors at both 33 °C and 37 °C. This seems to occur without extensive adaptation to the human host, as only a single non-synonymous mutation in the coding sequence of the HEF gene segment was identified among multiple samples. Intriguingly, the replication characteristics of IDV revealed much higher replication kinetics compared to Influenza C virus, despite sharing the cell tropism preference for ciliated cells. These results show that there is no intrinsic impairment for IDV propagation within the human respiratory epithelium.

For successful inter-species transmission, a virus needs to overcome several barriers before it can replicate efficiently in the new host species [41]. These barriers can be classified into three major groups; (i) viral entry through availability of the cellular receptor and proteases, (ii) viral replication and subversion of the host innate immune system followed by (iii) viral egress and release of infectious progeny virus. Our results clearly demonstrate that IDV fulfils most of these criteria for humans, as there is no fundamental restriction for viral replication and the sequential propagation of IDV within well-differentiated hAEC cultures from different donors did not lead to major genetic changes. Since only a single non-synonymous mutation was detected in the HEF protein among multiple samples. It is possible that this non-synonymous mutation results in some advantageous adaptation for the virus, however, since there is currently no reverse genetic system available for IDV, we cannot assess its functional relevance in our model at this time. Furthermore, we cannot assess whether IDV can be transmitted between humans with our model. Nonetheless, it has been demonstrated that IDV can be transmitted between both guinea pigs and ferrets, of which the latter is a surrogate model for assessing the transmission potential of emerging Influenza A viruses among humans [42,43,44,45,46]. This knowledge in combination with the detection of IDV in aerosols collected at an international airport, and the limited epidemiological data of IDV prevalence among humans, warrants the need for increased surveillance of IDV among humans [19,20].

At least two distinct genetic lineages are described for IDV, which have over 96% homology, from which the HEF glycoprotein (96.7 to 99.0% homology) is the most divergent of all 7 segments [2,10]. Because cattle are proposed as the main reservoir, we first selected to use only the prototypic D/bovine/Oklahoma/660/2013 strain, and therefore at that time did not include the prototypic D/swine/Oklahoma/1334/2011 strain as a representative of the other lineage. However, due to strict national import regulations for animal pathogens, we currently cannot assess whether both circulating lineages of IDV exhibit similar characteristics in human respiratory epithelium. Although, it is worth mentioning that IDV has been detected in a nasopharyngeal wash of a field worker with close contact to swine [18]. Suggesting that both lineages might exhibit similar characteristics in the human airway epithelium.

Influenza C virus is known predominantly as a common cold virus that is associated with mild upper respiratory tract infections in humans [39,40]. However, in our experiments we observed that ICV replicates efficiently in well-differentiated hAEC cultures at temperatures corresponding to both the upper and lower respiratory tract, albeit with a lower efficiency at 37 °C. This suggests that ICV in humans can replicate at lower regions in respiratory tract, which is in accordance with the detection of ICV in patients with lower respiratory tract infection [47]. Our results suggest that this is also possible for IDV that even had a higher replication efficiency at both temperatures in comparison to ICV. Similar observations have recently been made in undifferentiated primary nasal turbinate, tracheal and lung epithelial cells [48]. Nevertheless, whether the observed viral replication kinetics for ICV and IDV in our human in vitro surrogate model of the in vivo respiratory epithelium is recapitulated in those of swine and bovine origin remains to be evaluated. Especially, since both viruses have been demonstrated to be associated with respiratory infections in swine and cattle [1,2,49,50]. Furthermore, it is interestingly to note that in an experimental in vivo setting IDV can be found in both the upper and lower regions of the respiratory tract of cattle, whereas ICV has thus far only been detected in nasopharyngeal aspirates from the upper respiratory tract of cattle [11,49]. Therefore, it would be interesting to assess to which extend the different ambient temperatures in the upper and lower respiratory tract of both swine and cattle influences the viral replication efficiency of both ICV and IDV.

Both IDV and ICV utilize the 9-*O*-acetyl-*N*-acetylneuraminic acid as their receptor determinant for host cell entry [16,17]. We have shown that both viruses have a predominant affinity towards ciliated cells, suggesting that the distribution of this type of sialic acid is limited to ciliated cells within our in vitro model. This tropism is similar to what we previously observed for the human coronavirus OC43, from which it has been reported to also utilize the 9-*O*-acetyl-*N*-acetylneuraminic acid as receptor determinant [51,52]. Nonetheless, whether this cell tropism for both IDV and ICV corresponds to that of the in vivo airway epithelium remains to be determined. Although, we previously have demonstrated that the well-differentiated hAEC cultures recapitulates many characteristics of the in vivo airway epithelium, including receptor distribution [51,53].

In summary, we demonstrate that IDV replicates efficiently in an in vitro surrogate model of the in vivo respiratory epithelium. This shows that there is no intrinsic impairment of IDV propagation within the human respiratory epithelium and might explain why IDV-directed antibodies are detected among humans with occupational exposure to livestock.

## Figures and Tables

**Table 1:** Primer sequences for individual gene segment amplification of Influenza D virus (IDV).

Name Primer	Oligonucleotide Sequence (from 5′ to 3′)
Segment 1 Fw	CGG GTT ATT AGC AGT AGC AAG AGG ATT TTT TCA ATG TGC TTC AAA C
Segment 1 Rv	GGG GGG GCA TAA GCA GAG GAT GTC AC
Segment 2 Fw	CGG GTT ATT AGC AGT AGC AAG AGG ATT TTT CTG TTA TTA AAC AAC GC
Segment 2 Rv	GGG GGG GCA TAA GCA GAG GAT TTT ATA ACA ATG G
Segment 3 Fw	CGG GTT ATT AGC AGT AGC AAG GAG ATT TTT AAC ATT ACA AG
Segment 3 Rv	GGG GGG GCA TAA GCA GGA GAT TTA AAA ATG T
Segment 4 Fw	CGG GTT ATT AGC AGT AGC AAG GAG ATT TTT TCT AA
Segment 4 Rv	GGG GGA GCA TAA GCA GGA GAT TTT CAA AGA TG
Segment 5 Fw	CGG GTT ATT AGC AGT AGC AAG GAG ATT TTT TGT TAA ACA AGA CAA ACC AA
Segment 5 Rv	GGG GGG GCA TAA GCA GGA GAT TAT TAA GCA ATA
Segment 6 Fw	CGG GTT ATT AGC AGT AGC AAG AGG ATT TTT TCG
Segment 6 Rv	GGG GGG GCA TAA GCA GAG GAT ATT TTT GAC GC
Segment 7 Fw	CGG GTT ATT AGC AGT AGC AAG GGG TTT TTT C
Segment 7 Rv	GGG GGA GCA TAA GCA GGG GTG TAC AAT TTC AAT

**Table 2:** Summary of the detected nucleotide variants in serial-passaged IDV on hAEC culturs.

					Variant Detection Fraction in Total Number of Reads						
Gene	Position	Nucleotide Change	Variant	Amino Acid	Donor 1 at 33 °C	Donor 1 at 37 °C	Donor 2 at 33 °C	Donor 2 at 37 °C	Donor 4 at 33 °C	Donor 4 at 37 °C	Input
PB2	232	T > C	missense	Ile73Thr	0.030						
PB2	867	G > A	missense	Ala285Thr		0.995	0.521				
PB2	989	G > A	synonymous	Ala325Ala							0.031
PB2	2106	G > A	missense	Asp698Asn				0.087			
PB2	2273	C > T	synonymous	Ser753Ser	0.675						
PB1	325	G > A	synonymous	Lys100Lys				0.027			
PB1	328	G > A	missense	Met101Ile	0.023						
PB1	842	T > C	synonymous	Leu273Leu	0.142						
PB1	1144	A > G	synonymous	Lys373Lys							0.066
PB1	2002	G > C	synonymous	Thr659Thr				0.050			
PB1	2006	T > A	missense	Ser661Thr				0.054			
PB1	2033	T > C	synonymous	Leu670Leu				0.132			
PB1	2053	G > A	synonymous	Arg676Arg					0.083		
P3	385	C > A	missense	Phe121Leu				0.023			
P3	454	C > A	synonymous	Pro144Pro				0.021			
P3	538	C > A	missense	Phe172Leu				0.020			
P3	1615	G > A	synonymous	Val531Val	0.184						0.151
HE	276	G > A	synonymous	Ala84Ala							0.029
HE	770	A > G	missense	Asn249Ser	0.265	0.995	0.683		0.917	0.997	0.107
HE	841	R > A	missense	Val273Ile							0.793
HE	841	R > G	synonymous	Val273Val		0.998				0.999	0.203
HE	947	G > A	missense	Arg308Lys							0.036
HE	1145	A > G	missense	Asn374Ser				0.074			
HE	1585	G > A	missense	Gly521Ser							0.032
HE	1635	A > T	synonymous	Ala537Ala	0.067						
NP	432	C > A	missense	Phe135Leu				0.024			
NP	891	G > T	missense	Met288Ile				0.021			
NP	938	T > C	missense	Val304Ala				0.021			
NP	1335	G > A	synonymous	Glu436Glu				0.175			
NP	1446	C > A	missense	Phe473Leu				0.022			
NP	1596	G > T	synonymous	Val523Val				0.994			0.047
P42	108	G > A	missense	Arg27Lys							0.036
P42	772	G > T	missense	Leu248Phe				0.176			

**Table 3:** Summary of the total number of cells counted in ICV- and IDV-infected hAEC cultures.

	Influenza C Virus		Influenza D Virus	
*Total number of cells*	Infected (*n* = 6)	Mock (*n* = 3)	Infected (*n* = 6)	Mock (*n* = 3)
***Ciliated cells***	1078 (42.7%)	458 (49.1%)	1099 (48.3%)	584 (48.1%)
**N*on-ciliated cells***	1448 (57.3%)	474 (50.9%)	1174 (51.7%)	629 (51.9%)
***Total***	2526 (100%)	932 (100%)	2273 (100%)	1213 (100%)
*Total number of infected cells*				
***Ciliated cells***	82 (97.6%)	0 (0%)	94 (96.8%)	0 (0%)
**N*on-ciliated cells***	2 (2.4%)	0 (0%)	3 (3.2%)	0 (0%)
*Total*	84 (100%)	0 (0%)	97 (100%)	0 (0%)
***Percentage infected cells***	**3.3**	**0.0**	**4.3**	**0.0**

## Supplementary Materials

Supplementary materials can be found at https://www.mdpi.com/1999-4915/11/4/377/s1.
