# Supplementary material for: Determining the Replication Kinetics and Cellular Tropism of Influenza D Virus on Primary Well-Differentiated Human Airway Epithelial Cells
Source: Viruses. 2019 Apr 24;11(4):377. doi: 10.3390/v11040377 (PMC6521319; doi:10.3390/v11040377)
Supplement: Supplementary file 1 [file viruses-11-00377-s001.pdf]

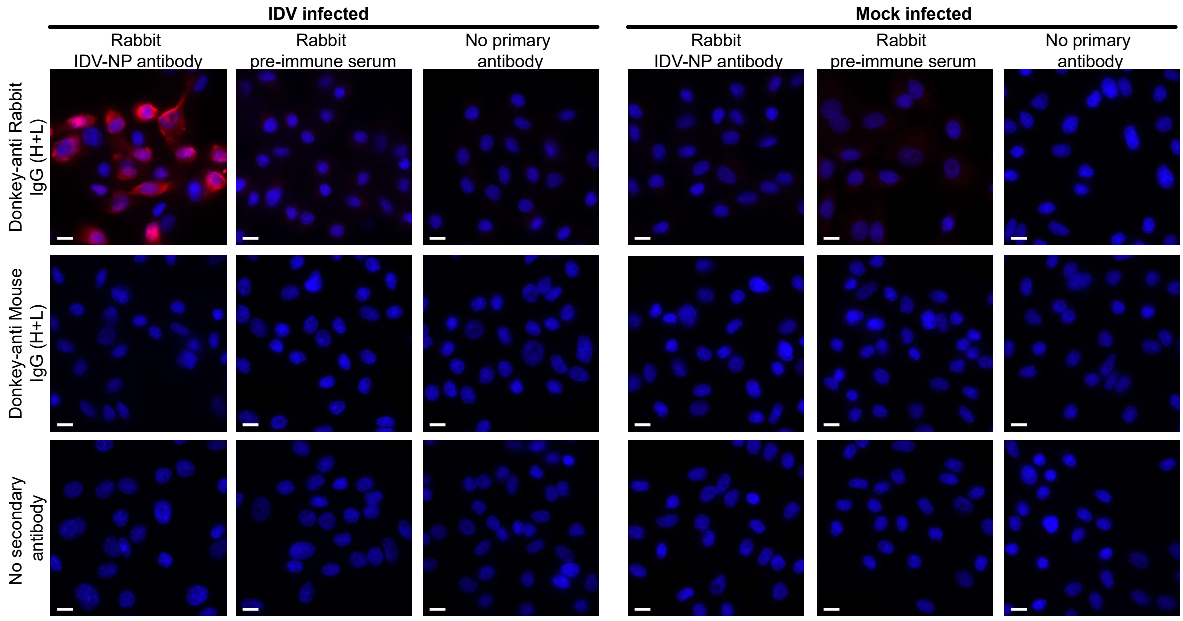


**Figure S1. Antibody specificity for the nucleocapsid (NP) protein of IDV.** To assess the specificity of the custom generated rabbit polyclonal antibody directed against the nucleoprotein (NP) of the prototypic D/bovine/Oklahoma/660/2013 strain, MDBK cells were mock-infected or infected with IDV at a MOI of 0.1 for 24 hours. Cells were incubated with either the rabbit-derived antibody against the NP of IDV, corresponding pre-immunization rabbit serum or no primary antibody at all. The samples were counterstained with either Alexa Fluor® 647-labeled donkey anti-Rabbit IgG (H+L), Alexa Fluor® 647-labeled donkey anti-Mouse IgG (H+L) or no secondary antibody at all to visualize the specificity of the primary and secondary antibodies (red). In addition, all samples were counterstained using 4',6-diamidino-2-phenylindole (DAPI) to visualize the nuclei (blue). Magnification 60x, the scale bar represents 10 micrometers.

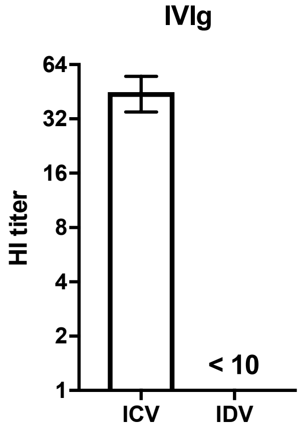


**Figure S2. Detection of human antibodies directed to IDV and ICV in IVIg.** To assess the presence of antibodies directed towards IDV and ICV a hemagglutination inhibition assay was performed using 8 HA units per 50 µL of virus and 2-fold serial dilution of receptor-destroying enzyme pretreated Intravenous Immunoglobulins (IVIg). The resulting HI-titer (y-axis) against ICV (white) and IDV (black) is shown as the mean and SD from duplicates from four independent experiments.
